# Intermittent hypoxia regulates vasoactive molecules and alters insulin-signaling in vascular endothelial cells

**DOI:** 10.1038/s41598-018-32490-3

**Published:** 2018-09-20

**Authors:** Pragya Sharma, Yu Dong, Virend K. Somers, Timothy E. Peterson, Yuebo Zhang, Shihan Wang, Guangxi Li, Prachi Singh

**Affiliations:** 1 Department of Cardiovascular Medicine, Mayo Clinic, Rochester, MN 55905 USA; 2 Guang’anmen Hospital, China Academy of Chinese Medical Sciences, Beijing, 100053 China; 3 Department of Neurosurgery Research, Mayo Clinic, Rochester, MN 55905 USA; 4 Department of Pulmonary and Critical Care Medicine, Rochester, MN 55905 USA

## Abstract

Vascular dysfunction and insulin resistance (IR) are associated with obstructive sleep apnea (OSA), which is characterized by frequent episodes of nocturnal intermittent hypoxia (IH). While it is recognized that the balance between vasoconstrictive (endothelin-1) and vasodilatory molecules (nitric oxide, NO) determine vascular profile, molecular mechanisms contributing to vascular dysfunction and IR in OSA are not completely understood. Caveolin-1 is a membrane protein which regulates endothelial nitric oxide synthase (eNOS) activity which is responsible for NO generation and cellular insulin-signaling. Hence, we examined the effects of IH on caveolin-1, eNOS, and endothelin-1 in human coronary artery endothelial cells in the context of IR. Chronic 3-day IH exposure up-regulated caveolin-1 and endothelin-1 expression while reducing NO. Also, IH altered insulin-mediated activation of AKT but not ERK resulting in increased endothelin-1 transcription. Similarly, caveolin-1 overexpression attenuated basal and insulin-stimulated NO synthesis along with impaired insulin-dependent activation of AKT and eNOS, with no effect on insulin-stimulated ERK1/2 phosphorylation and endothelin-1 transcription. Our data suggest that IH contributes to a vasoconstrictive profile and to pathway-selective vascular IR, whereby insulin potentiates ET-1 expression. Moreover, IH may partly mediate its effects on NO and insulin-signaling via upregulating caveolin-1 expression.

## Introduction

Obstructive sleep apnea (OSA) is a common sleep breathing disorder, and is an acknowledged risk factor for cardiovascular disease^1^. OSA causes transient cessation of breathing during sleep with repetitive episodes of hypoxemia-reoxygenation leading to chronic intermittent hypoxia (IH) exposure^1^. Endothelial dysfunction is characterized by decreased bioavailability of endothelium-derived nitric oxide (NO), and is highly prevalent in OSA^2^. Impaired endothelial function is also an important early clinical marker for predicting atherosclerosis and future cardiovascular events. In addition, there is a distinct association of OSA with development of insulin resistance^3^ (IR) and hypertension^1^. Treatment with continuous positive airway pressure therapy improves vascular function, hypertension as well as insulin-sensitivity^1,4^. This suggests a role of chronic IH in eliciting endothelial dysfunction and IR. However, the molecular mechanisms underlying this association are not completely elucidated.

NO and endothelin-1 (ET-1) are vasoactive compounds expressed in endothelial cells. NO has a vasodilatory effect on blood vessels while ET-1 is a potent vasoconstrictor^5^. In endothelial cells, NO is generated by endothelial nitric oxide synthase (eNOS) activity, which is regulated by caveolin-1 (cav-1), by directly binding to and blocking the active site of eNOS^6^. Additionally, cav-1 also regulates insulin signaling^7^. Considering the dual role of cav-1 in regulating both eNOS and insulin-signaling, we sought to identify and define the role of cav-1 in regulating endothelial NO synthesis and insulin signaling in response to chronic IH using cultured human coronary artery endothelial cells (HCAEC). We tested the hypothesis that IH increases cav-1 and mediates vascular dysfunction and IR with consequent decreases in NO, increases in ET-1, and selectively impaired insulin signaling. Furthermore, we hypothesized that IH dependent effects on vasoactive compounds and insulin cellular signaling may be mimicked by cav-1 overexpression.

## Results

### IH decreases NO generation while increasing cav-1 and ET-1 expression

**Figure 1:**
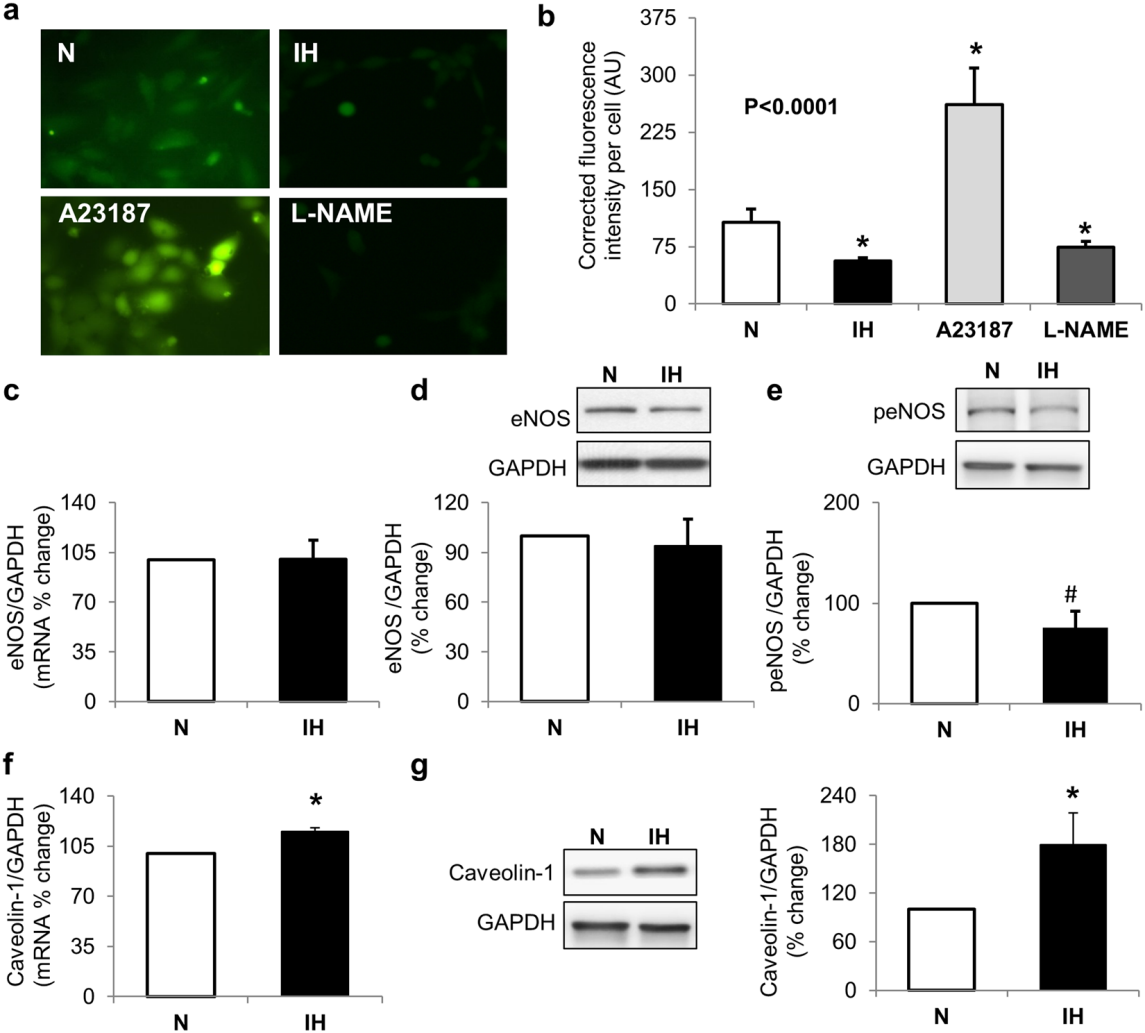
Intermittent hypoxia alters NO generation. Representative images (**a**) and graph (**b**) showing decreased NO generation (green fluorescence) in cells exposed to intermittent hypoxia. A23187 was used as a positive control and L-NAME was used as a negative control. Graph and representative Western Blots showing no changes in eNOS mRNA (**c**), total protein (**d**), and phosphorylated protein (**e**). Graph and representative Western blots showing increases in caveolin-1 mRNA (**f**) and protein (**g**). Data are presented as mean ± SEM from at least 3 independent experiments. P values were determined using Wilcoxon Rank Sums test. For pairwise comparison (**b**), Wilcoxon method was used. *is p < 0.05, ^#^is p < 0.07 compared to normoxia control. N: normoxia (white bars); IH: intermittent hypoxia (black bars); eNOS: endothelial nitric oxide synthase.

**Figure 2:**
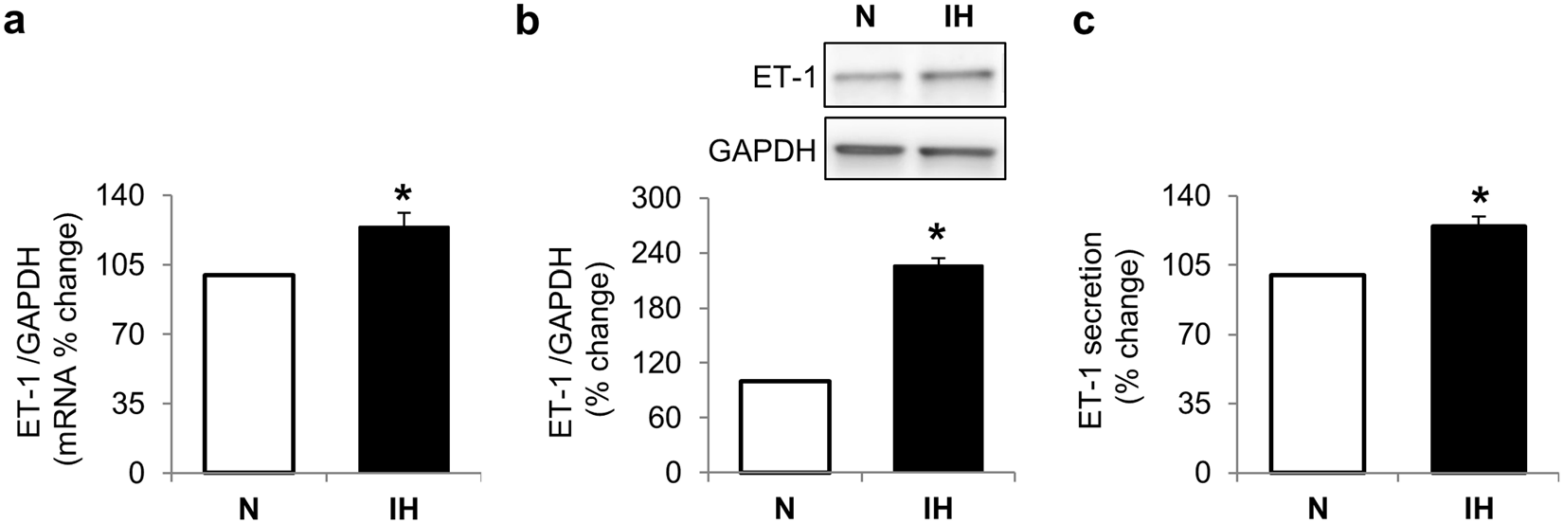
Intermittent hypoxia upregulates endothelin-1. Graphs and representative Western blot showing intermittent hypoxia mediated changes in endothelin-1 mRNA (**a**), intracellular protein (**b**) and secreted protein (**c**). Data are presented as mean ± SEM from at least 3 independent experiments. P values were determined using Wilcoxon Rank Sums test. *is p < 0.05 compared to normoxia control. N: normoxia (white bars); IH: Intermittent hypoxia (black bars); ET-1: endothelin-1.

To understand the role of IH in endothelial cells, we treated HCAEC with IH or normoxia. IH reduces tonic NO generation (p = 0.01, Fig. 1a,b). The IH mediated decreases in NO were similar to that observed by treatment with L-NAME, a known eNOS inhibitor. At the same time, IH does not alter eNOS mRNA (p = 0.28) and protein expression (p = 1.0) but a trend for reduced expression of active phosphorylated eNOS (ser1177) was apparent (p = 0.065, Fig. 1c–e). Next, we examined the effect of IH on cav-1 expression and show that IH increases cav-1 mRNA (p = 0.04) and protein (p = 0.01, Fig. 1f,g). IH also increases ET-1 mRNA (p = 0.01), intracellular protein expression (p = 0.02) and ET-1 secretion in conditioned media (p = 0.002, Fig. 2).

### IH partially alters insulin cellular signaling pathways

**Figure 3:**
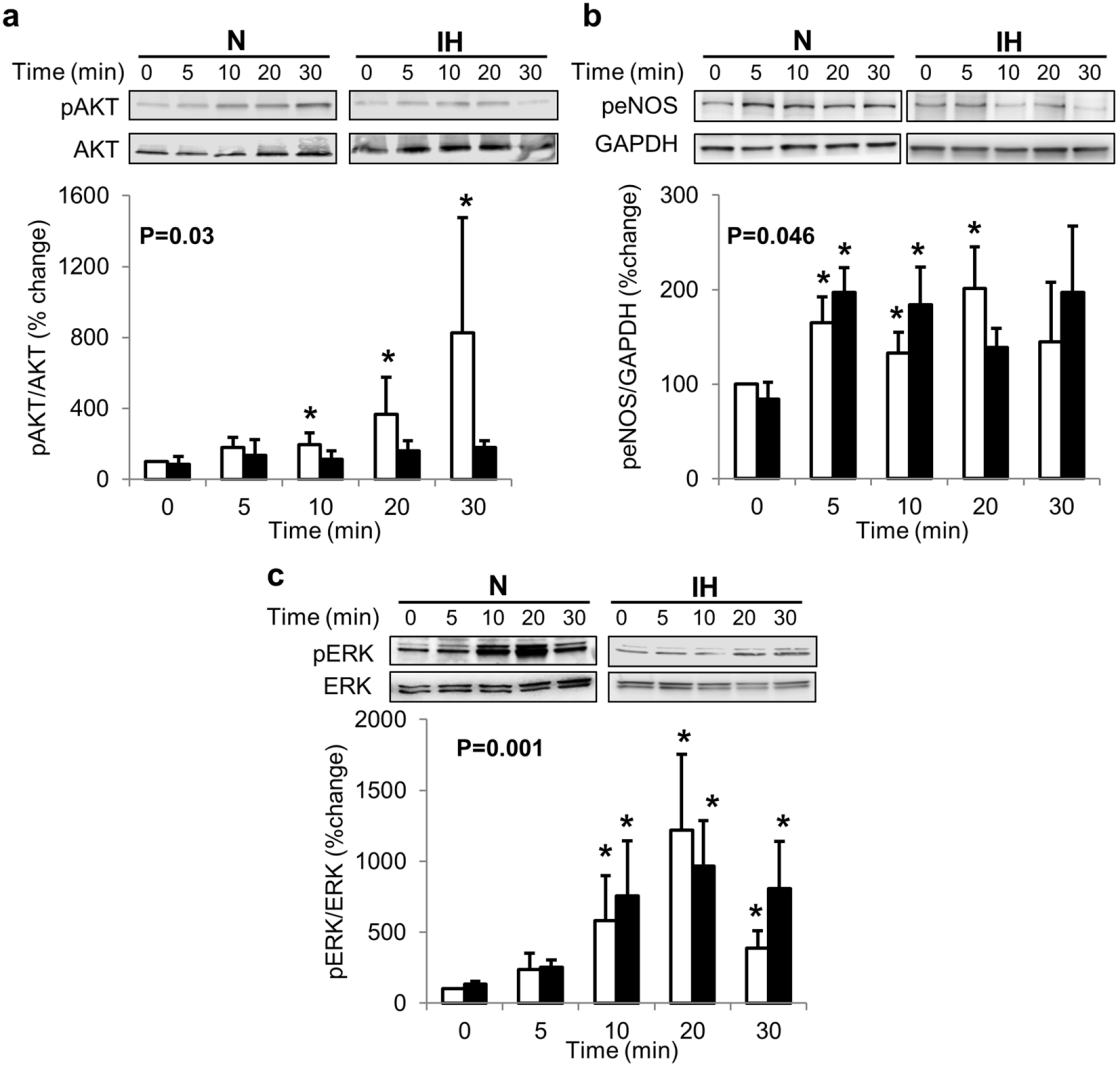
Intermittent hypoxia alters insulin dependent cellular signaling pathways. Representative Western blots and graphs showing intermittent hypoxia induced changes in insulin mediated phosphorylation of AKT (**a**), eNOS (**b**) and ERK (**c**). Data are presented as mean ± SEM from at least 3 independent experiments. Overall P values were determined using Wilcoxon Rank Sums test. For pairwise comparison, Wilcoxon method was used. *is p < 0.05 as compared to 0 time point for Normoxia (N) or intermittent hypoxia (IH) group respectively. White bars depict data from N group, black bars depict data from IH group.

**Figure 4:**
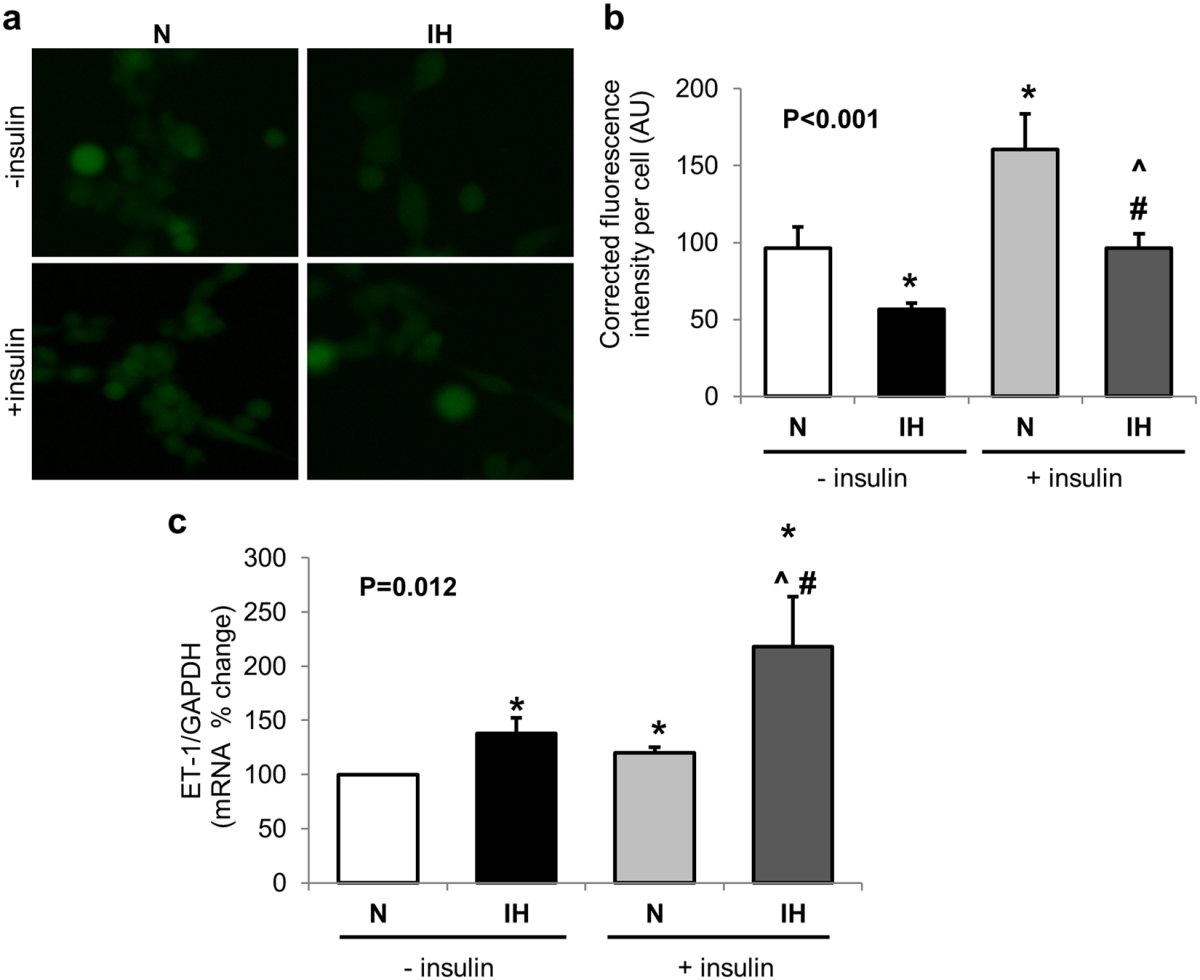
Chronic 3-day exposure to intermittent hypoxia alters insulin-mediated cellular actions. Representative images (**a**) and graph (**b**) showing insulin-dependent NO generation (green fluorescence) in cells exposed to IH. Graph showing increased insulin-mediated transcription of endothelin-1 in cells exposed to intermittent-hypoxia (**c**). Data are presented as mean ± SEM from at least 3 independent experiments. Overall P values were determined using Wilcoxon Rank Sums test. For pairwise comparison, Wilcoxon method was used. *is p < 0.05 compared to normoxia control w/o insulin. ^#^p < 0.05 compared to IH w/o insulin, ^p < 0.05 compared to N with insulin. N: normoxia; IH: Intermittent hypoxia; ET-1: endothelin-1.

OSA may potentiate development of insulin-resistance^8^; therefore we examined the effects of chronic IH on insulin cellular actions. Insulin mediated activation of AKT (p = 0.02), eNOS (p = 0.045) and ERK (p = 0.005) cellular signaling pathways was observed in control cells grown in continuous normoxia (Fig. 3). However, insulin was unable to phosphorylate AKT (p = 0.19) in cells exposed to IH (Fig. 3a). Insulin-dependent phosphorylation of eNOS was apparent in cells exposed to IH (p = 0.09, Fig. 3b) especially after 5 and 10 min of insulin treatment. Similar to cells grown in normoxia, insulin-mediated phosphorylation of ERK (p = 0.013) was evident in cells exposed to chronic IH (Fig. 3c). Additionally, insulin stimulated NO generation in cells exposed to normoxia as well as IH. However, even after insulin stimulation, NO levels remained lower in cells exposed to IH (p < 0.001, Fig. 4a,b). Importantly, insulin stimulated ET-1 transcription was enhanced in cells exposed to IH (p = 0.006, Fig. 4c).

### Differential effects of cav-1 overexpression on cellular insulin actions

**Figure 5:**
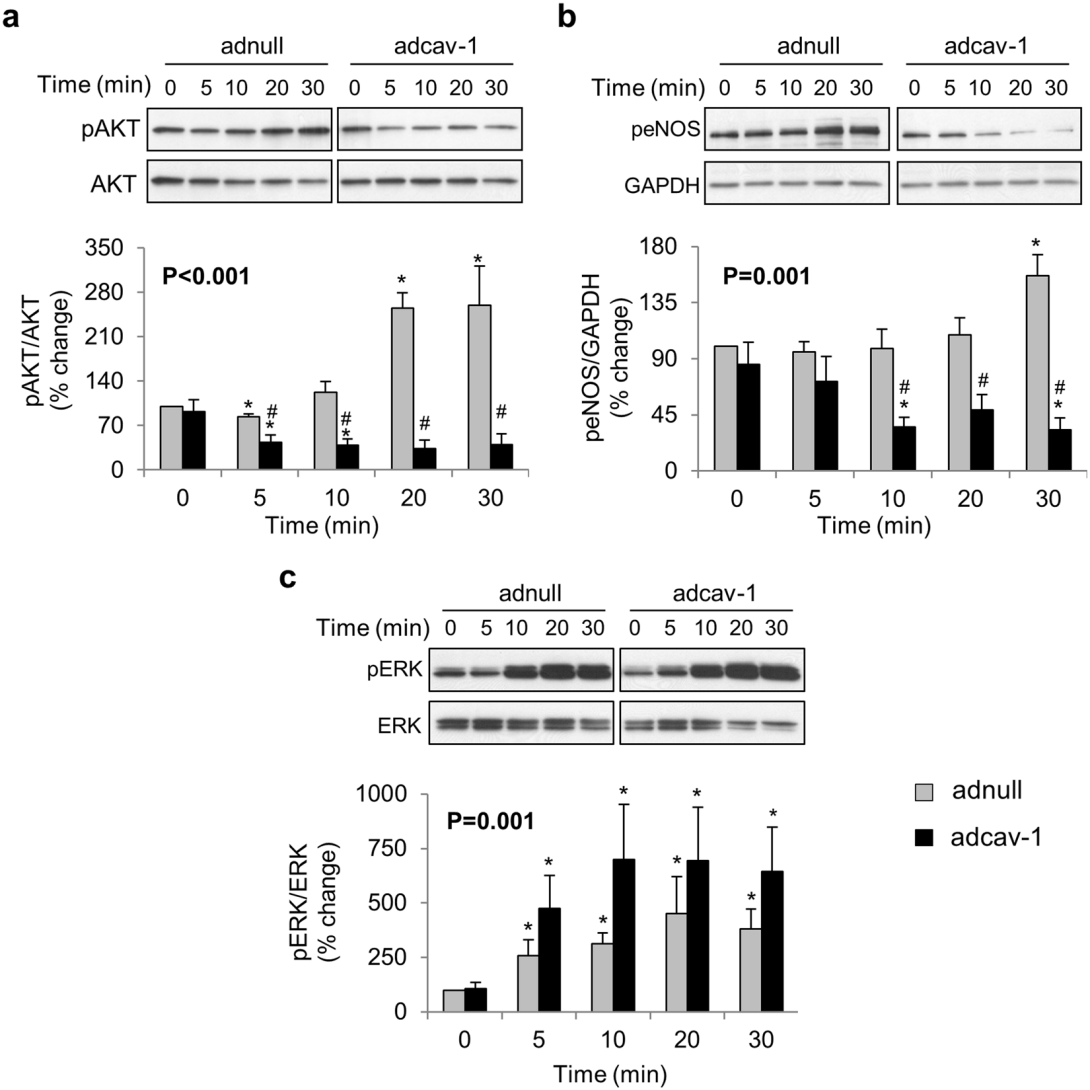
Caveolin-1 (cav-1) expression alters insulin cellular signaling. Cav-1 overexpressing (adcav-1, black bars) or control (adnull, light gray bars) cells were treated with insulin (100 nM) for increasing time periods. Representative western blot and densitometry graph showing that cav-1 overexpression causes attenuation of insulin dependent phosphorylation of AKT (**a**) and eNOS (**b**). Insulin-mediated ERK phosphorylation is not altered by caveolin-1 expression (**c**). Data are mean ± SEM. Overall P values were determined using Wilcoxon Rank Sums test. For pairwise comparison, Wilcoxon method was used. *p ≤ 0.05 compared to 0 time point adnull or adcav-1 treated cells respectively. #p < 0.05 compared to ad-null treated cells at the same time point.

**Figure 6:**
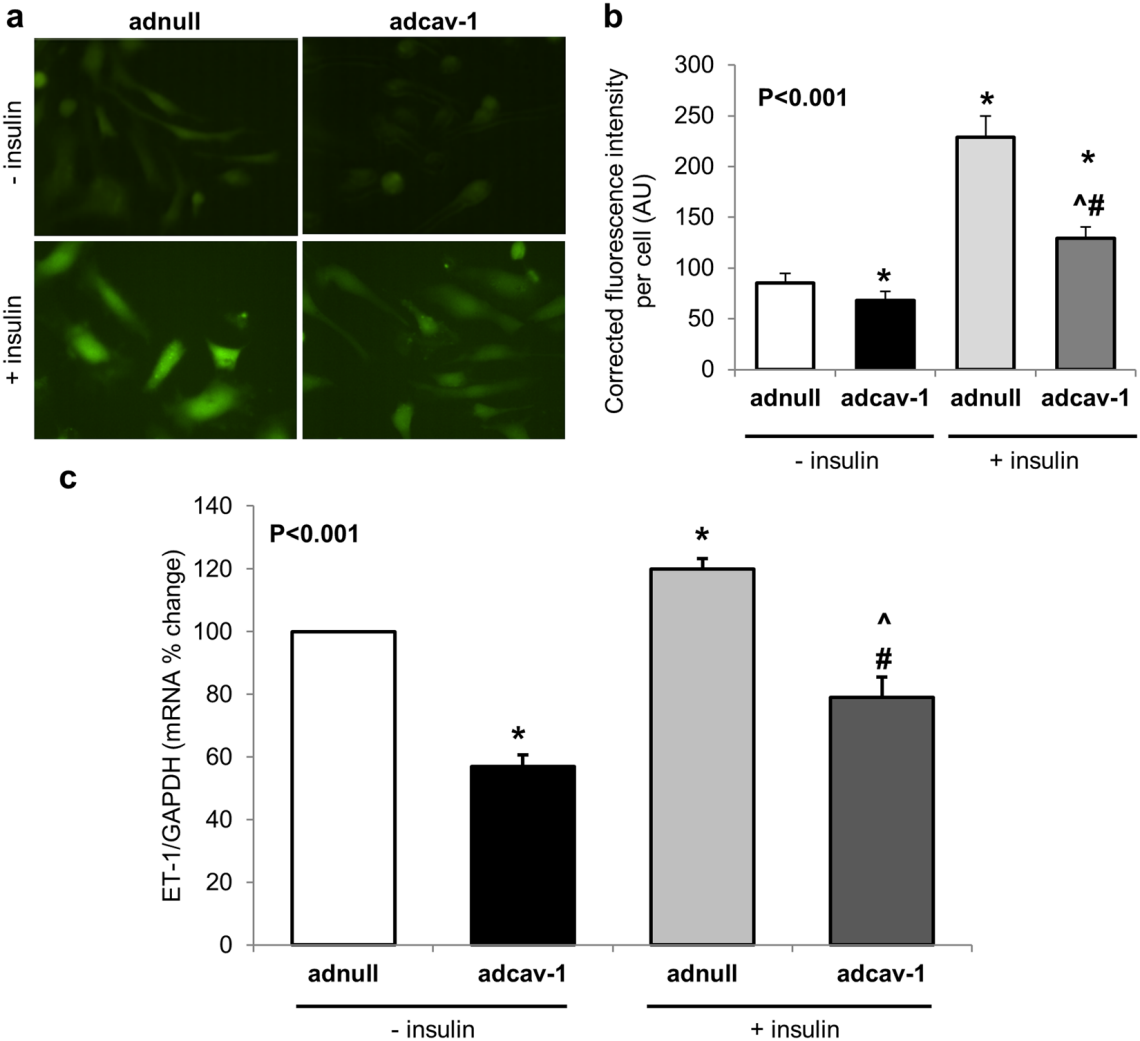
Caveolin-1 overexpression attenuates insulin mediated cellular actions. Representative images (**a**) and graph (**b**) showing NO generation (green fluorescence) in basal and insulin stimulated conditions in control (adnull) and caveolin-1 overexpressing (adcav-1) cells. Graph (**c**) showing stimulation of ET-1 mRNA expression by insulin (100 nM) in control (ad-null) and cav-1 overexpressing cells. Data are mean ± SEM. Overall P values were determined using Wilcoxon Rank Sums test. For pairwise comparison, Wilcoxon method was used. *p < 0.05 compared to adnull cells w/o insulin, #p < 0.05 compared to adcav-1 cells w/o insulin and ^p < 0.05 compared to adnull cells with insulin.

Cav-1 regulates insulin-signaling^7^ and IH upregulates cav-1 expression; therefore we investigated the effects of increased cav-1 on insulin-dependent activation of signaling pathways including AKT and ERK1/2, and downstream activation of eNOS in HCAEC. Cav-1 overexpression impaired insulin-dependent activation of AKT (p = 0.02, Fig. 5a) and eNOS (p = 0.03, Fig. 5b). Of note, 30 min of insulin exposure was able to increase AKT phosphorylation in control adnull cells. Also, attenuation of insulin-dependent signaling by increased cav-1 is selective since insulin-stimulated phosphorylation of ERK1/2 (p = 0.02, Fig. 5c) was not affected in cells treated with adcav-1. Furthermore, cav-1 overexpression attenuated basal and insulin-stimulated NO synthesis (p < 0.0001, Fig. 6a,b). Insulin dependent upregulation of ET-1 mRNA transcription is not altered by increases in cav-1 expression (p < 0.001, Fig. 6c).

## Discussion

In this study we demonstrate for the first time that IH alters insulin-signaling and stimulates cav-1 expression which may together promote changes in HCAEC consistent with vasoconstriction. These findings have important implications for understanding mechanisms contributing to the increased risk of diabetes and hypertension in patients with OSA.

Endothelial dysfunction, high blood pressure, and IR in OSA likely contribute to the increased cardiovascular risk in OSA patients^1^. It has been reported that reduced plasma levels of vasodilator NO correlate with the severity of IH^9^, while plasma levels of vasoconstrictor ET-1 are elevated in OSA^10^. These observations suggest involvement of IH in regulation of vascular function in OSA, but the underlying molecular mechanisms are not known. Our study is the first to examine the role of IH in mediating changes in cav-1, NO, ET-1, and insulin-signaling in human coronary artery endothelial cells.

Cav-1 has an important role in regulating NO-dependent vasodilation in endothelial cells. eNOS, as a source of bioavailable NO in the vascular endothelium, plays a critical role in maintaining vascular homeostasis by exerting vasodilatory, anti-inflammatory and anti-thrombotic effects while actively promoting endothelial repair, regeneration and vascularization^11^. Over-expression of cav-1 inhibits eNOS activity, endothelial barrier function, and angiogenic responses to exogenous VEGF and tissue ischemia^12^. On the other hand, in cav-1^(−/−)^ knock-out mice, constitutive activation of eNOS is reported to cause microvascular hyperpermeability, which is reversed by L-NAME (specific NOS inhibitor) injection^3^. This suggests that cav-1 could potentially have a role in modulating endothelial function in OSA via IH. In our *in-vitro* model of OSA we demonstrate that cav-1 expression increased after IH exposure along with a decline in NO. Moreover, IH had a stimulatory effect on the expression as well as secretion of ET-1, which is known to have vasoconstrictive properties^5^. IH mediated increases in ET-1 are consistent with observations from several animal models of OSA^13–15^. Furthermore, both cav-1 and ET-1 have been shown to be modulated by transcription factor Hif-1^14,16^, which is known to play an important role in IH as well^17^. Therefore, it is likely that Hif-1 represents a common underlying mechanism responsible for altering both cav-1 and ET-1in IH and may be targeted to attenuate detrimental effects of IH on vascular function.

In addition to the role of cav-1 in regulating eNOS activity, cav-1 is also known to alter insulin signaling^18^. Cav-1 is essential to stabilize the insulin receptor and is known to differentially modulate insulin-dependent cellular signaling pathways^7,18^. Furthermore, cav-1 has been shown to be important for insulin uptake in vascular endothelial cells^19^. Therefore, it is likely that IH mediates its effects on insulin signaling in part via increasing cav-1 expression. In addition to regulating metabolism, insulin is also critical for regulation of endothelial vasomotor function by increasing vascular endothelial NO production. At the cellular level, this regulation of endothelium-mediated vasoactivity is actually a balance between phosphatidylinositol 3-kinase- (PI3K)-dependent insulin-signaling pathways known to regulate endothelial NO production and mitogen activated protein kinase (MAPK)-dependent insulin-signaling pathways that regulate the secretion of the vasoconstrictor ET-1^20^. The normally functioning endothelium keeps ET-1 and NO in balance. Based on work by us^21^ and others^22^, this balance between vasodilator and vasoconstrictor responses to insulin is altered with age and with IR. We have previously shown that insulin promotes vasodilation in healthy young adults but causes vasoconstriction in healthy elderly individuals^21^, suggesting an attenuation of the vasodilator effect and/or a potentiation of the vasoconstriction action of insulin in the elderly. It was speculated that the vasoconstrictor response to insulin might further potentiate IR in the elderly. Similar to aging, with IR, the PI3K-NO pathway is reduced while the MAPK-ET-1 pathway is unaffected or stimulated, leading to endothelial dysfunction in animal models or humans^20^. We show that increased cav-1 expression alters the balance between insulin-mediated PI3K-NO and MAPK-ET1 signaling such that insulin contributes to a more vasoconstrictive profile. Furthermore, while cav-1 binds to and directly regulates eNOS activity via interference with Ca^2+^-dependent NO synthesis, insulin activates eNOS in a Ca^2+^-independent manner via AKT^6,23^. There is evidence that activation of AKT may exert anti-atherogenic effects through posttranscriptional activation of eNOS by phosphorylation at the ser^1177^ amino acid residue^24^. Whether cav-1 has a role in regulation of insulin-mediated increases in NO generation is not known. We demonstrate that increased cav-1 expression attenuates insulin-dependent NO synthesis in endothelial cells (Fig. 6). Our results indicate that cav-1 selectively impairs insulin-dependent signaling with preferential inhibition of AKT versus ERK1/2 signaling. Vasoconstrictor and mitogenic effects of ET-1 may be implicated in increased cardiovascular risk in patients with OSA. The findings related to partial impairment of insulin cellular signaling pathways are consistent with previous reports in different cell types^7,25,26^. Interestingly, in contrast to cav-1 overexpressing cells which impaired insulin dependent activation of AKT and eNOS, chronic exposure to IH only altered insulin-mediated phosphorylation of AKT while insulin mediated activation of eNOS and NO generation was intact. Furthermore, IH exposure enhanced insulin-mediated ET-1 transcription. Intriguingly, insulin has been shown to activate eNOS via AMPK pathway in human platelets^27^. These findings warrant further in-depth investigation of cellular signaling pathways being altered with IH. Nevertheless, our study provides a novel molecular mechanistic basis for the insulin-mediated changes in vascular tone and development of selective insulin-resistance with IH exposure.

The strength of our study is in the *in-vitro* IH model which has been previously shown to mimic molecular changes seen in OSA subjects^28^ and use of human coronary artery endothelial cells which are relevant to development of atherosclerosis. Furthermore, we have used both eNOS phosphorylation and DAFDA based NO detection to inform on eNOS activity in the presence of appropriate positive and negative controls. Our study has several limitations including those related to *in-vitro* study design, which merits further investigation in OSA patients. To conclusively prove the role of cav-1 in IH-mediated changes in eNOS and insulin signaling, cav-1 silencing experiments may be envisaged. However, previous studies have shown that the lack of cav-1 can itself increase basal eNOS activity and impair insulin-signaling as well^3,29^. Therefore, considering the requirement for optimal cav-1 levels to regulate eNOS and facilitate insulin signaling, it is not feasible to undertake cav-1 silencing experiments in conditions of IH and we restricted our studies only to examining the contribution of increased cav-1 on basal and insulin-stimulated eNOS activity in normoxic conditions.

In conclusion, we show that IH promotes imbalance between vasodilatory and vasoconstrictive molecules in vascular endothelial cells. The effects of IH are further potentiated by enhanced insulin-stimulated ET-1 transcription in cells treated with IH. Furthermore, IH induces cav-1 expression. Increased cav-1 expression may in-turn selectively alter insulin-signaling so as to promote vasoconstrictive responses by attenuating NO generation via the AKT pathway but not altering insulin-dependent stimulation of ET-1 transcription via the ERK1/2 pathway. Our findings identify a potential cav-1 dependent mechanism through which OSA may contribute to insulin-resistance as well as a vasoconstrictive profile. It would be of clinical interest to determine whether increases in cav-1 are also evident in OSA patients and can be attenuated by continuous positive airway pressure treatment. Furthermore, it would be important to determine if increases in cav-1 are also evident in other tissues such as liver, pancreas and heart. Nevertheless, our studies provide a molecular basis for potentially targeting vascular cav-1 to improve insulin-signaling and vascular profile in OSA.

## Materials and Methods

### Cell Culture and Treatments

Human coronary artery endothelial cells (HCAEC) and cell culture reagents were obtained from Lonza, Clonetech (Mountain View, CA). Cells were grown in endothelial growth media 2 (EGM-2) supplemented with growth factors and 5% FBS. All the experiments were performed at 3–5 passages with 70–80% confluence^30^. Intermittent hypoxia was achieved by cycles of 30 min 0.1% O_2_ + 5% CO_2_ and 30 min 21% O_2_ + 5% CO_2_ repeated 9 times each day using Oxycycler (Biospherix, Lacona, NY) through regulated expulsion of oxygen and nitrogen^28^. Cells grown in continuous 21% O_2_ + 5% CO_2_ were used as normoxic controls for these experiments. This *in-vitro* model of IH has been previously demonstrated to mimic molecular changes observed in OSA patients^28^. Cells were treated with IH for 3 days for protein and 1 day for RNA analyses. The length of IH exposure is based on experiments examining changes in cav-1 over period of 1 to 5 day (data not shown). IH exposed cells were either used for NO staining and insulin-signaling experiments or frozen immediately for protein or mRNA analysis. To achieve maximal activation of insulin signaling pathways, 100 nM insulin concentration was used^31^. Visual assessment of cells was undertaken at each step to ensure viability of the cells and optimal data quality.

### Cav-1 Overexpression

To assess the effect of cav-1 overexpression in HCAEC, cells were infected with adenovirus expression vector encoding cav-1 gene (adcav-1; Vector Biolabs, Malvern, PA)^30^. Cells infected with null adenovirus (adnull) were used as a control. Cells were serum-starved overnight and were infected with adenovirus (MOI 40) for 24 hrs. Infected cells were allowed to grow for another 24 hrs. in complete growth medium. To ascertain whether cav-1 overexpression has an effect on insulin-dependent signaling, adcav-1 cells were serum-starved overnight and treated with 100 nM Insulin for different time points (0–30 min, 6 hrs).

### Protein Analyses

Intracellular protein expression in treated cells was determined by Western blot analysis. Primary antibodies used include eNOS (#5880 S, 1:1000, Cell Signaling, Danvers, MA), peNOS (#9570, Ser^1177^, 1:1000, Cell Signaling), cav-1(#610407, 1:2000, BD transduction Laboratories, San Diego, CA),ET-1 (#ab117757, 1:2000, Abcam Inc., Cambridge, MA), pERK (#9101, Thr^202^/Tyr^204^, 1:1000, Cell Signaling), ERK(#9102, 1:1000, Cell Signaling), pAKT (#9275, 1:1000, Cell Signaling), AKT(#9272, Ser^473^, 1:1000, Cell Signaling), and GAPDH(#2118, 1:2500, Cell Signaling). Complete Western blots for the representative images are presented in supplementary material. The optical density of the band was measured using ImageJ software (NIH). The protein expression levels were normalized to GAPDH or total protein (for experiments measuring phosphorylated protein) and represented as percent increase as compared to relative controls. Secreted ET-1 levels were assessed in the condition medium using quantikine ELISA kit (R&D, Minneapolis, MN) as per manufacturers’ instruction. For ELISA, all the samples and standards were run in duplicates. The results were expressed as percentages relative to the respective controls.

### mRNA Analysis

Total RNA was isolated from treated cells by Purelink RNA mini kit and reverse transcribed to cDNA using high-capacity cDNA transcription kit. Commercially available validated TaqMan gene-specific expression assays were used to quantify the transcripts for cav-1, eNOS, ET-1 and GAPDH (endogenous control) in the cDNA library. RTPCR was performed on iCycler IQ real time PCR detection system (Biorad, Hercules, CA). RT-PCR reactions were performed according to manufacturer’s instructions. All samples and standards were run in triplicate and GAPDH was used as internal control. Relative standard curves from known dilutions of cDNA from HCAEC were used to semi-quantify the gene expression. The gene expression was expressed as normalized ratio and percent change compared to relative control group.

### Nitric oxide Detection

To determine the effects of IH and cav-1 overexpression on basal and insulin-stimulated NO production, cells grown in IH or normoxia were treated with insulin (100 nM), L-NAME (negative control, 5 mM), and A23187 (positive control, 5 µM) in HBSS buffer containing L-arginine (100 µM) for 1 hour. Subsequent to this treatment, cells were washed with HBSS and incubated with cell permeable fluorescent NO indicator 4- amino- 5- methylamino-2′, 7′-difluorescein diacetate (DAF-FM-Da, Life Technologies, Grand Island, NY) (1 µM, 30 min) in HBSS buffer containing L-arginine for 30 min. Prior treatment with insulin or other controls were repeated for 30 min and production of NO was visualized by emission of green light (515 nm) upon excitation at 489 nm using fluorescence imaging system (X-cite 120). Images were captured using a Nikon digital camera attached to an inverted light microscope at 40X magnification. Fluorescence intensity was quantified using ImageJ (NIH) software for at least 25 cells per treatment during each experiment. Corrected total cell fluorescence was calculated by subtracting the integrated fluorescence density value obtained for the cell with the multiple of area of the selected cell and mean fluorescence of background readings. These corrected total cell fluorescence values were used for further analysis. Each experiment was independently repeated at least three times.

### Statistical Analysis

Data are presented as Mean ± SEM. The differences between the groups were assessed using nonparametric Wilcoxon Rank Sums test. For experiments with more than two groups, Wilcoxon method was used for paired comparisons. JMP software from SAS Institute Inc version 10.0 was used for all analysis and P < 0.05 was considered statistically significant. All western blots and *in vitro* experiments were repeated independently on at least three occasions.

## Electronic supplementary material

Supplementary Images

## Data Availability

The data generated during and/or analyzed during the current study are available from the corresponding author on reasonable request.
