# Supplementary material for: Intermittent hypoxia regulates vasoactive molecules and alters insulin-signaling in vascular endothelial cells
Source: Sci Rep. 2018 Sep 20;8:14110. doi: 10.1038/s41598-018-32490-3 (PMC6148090; doi:10.1038/s41598-018-32490-3)
Supplement: Supplementary file 1 — Supplementary Images [file 41598_2018_32490_MOESM1_ESM.pdf]

Intermittent hypoxia regulates vasoactive molecules and alters insulin-signaling in vascular endothelial cells

Pragya Sharma, Yu Dong, Virend K. Somers, Timothy E. Peterson, Yuebo Zhang, Shihan Wang, Guangxi Li, Prachi Singh

Fig 1d

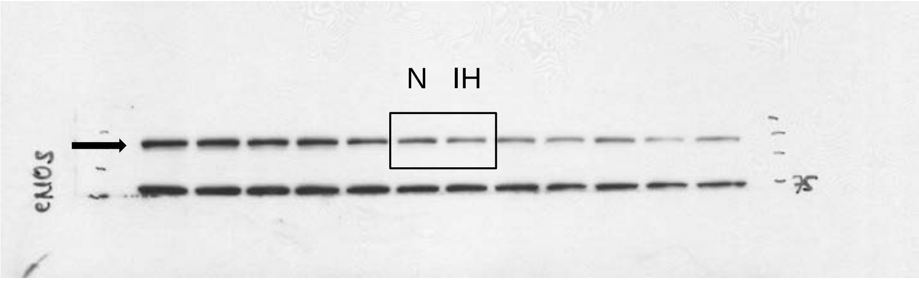

Fig 1d

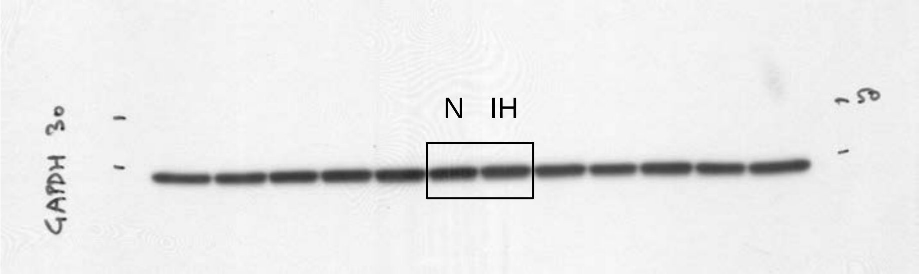

Fig 1e, peNOS

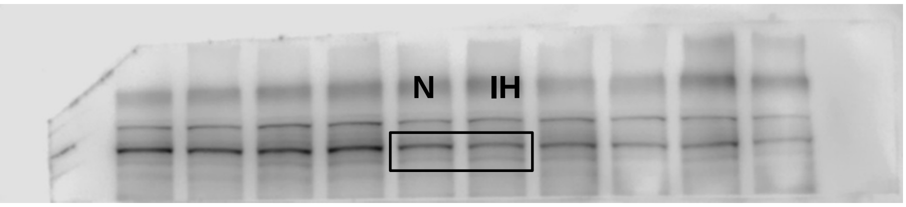

Fig 1e, 2b, GAPDH

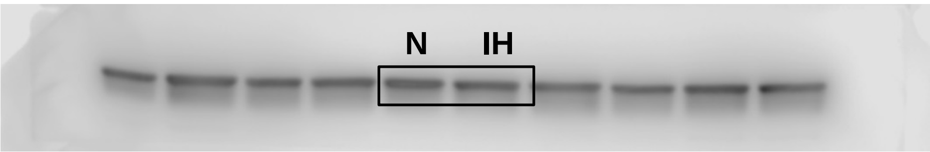

Fig 2b, ET-1

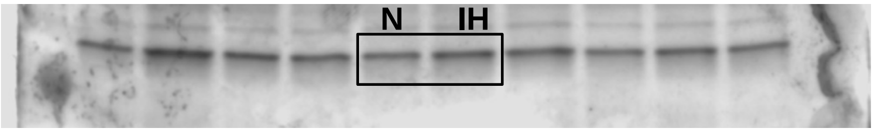

Fig 1g, Cav-1

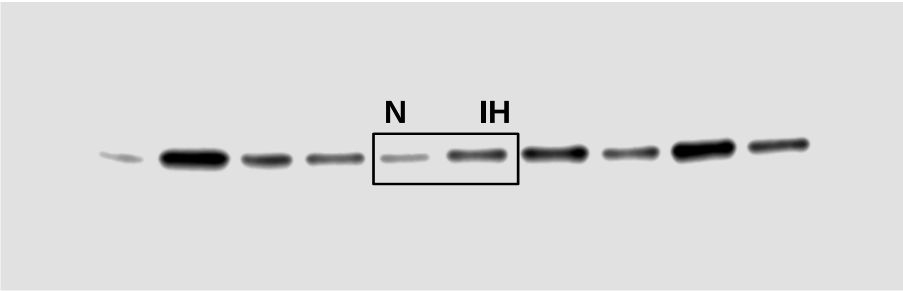

Fig 1g, Fig 3b, GAPDH

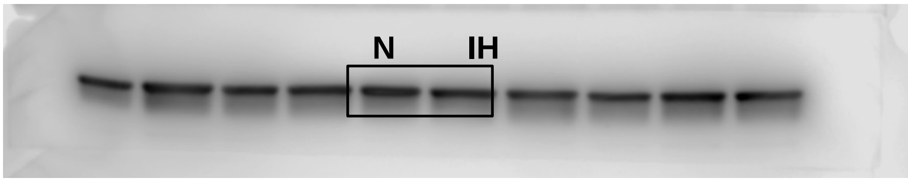

Fig 3b, peNOS (min.)

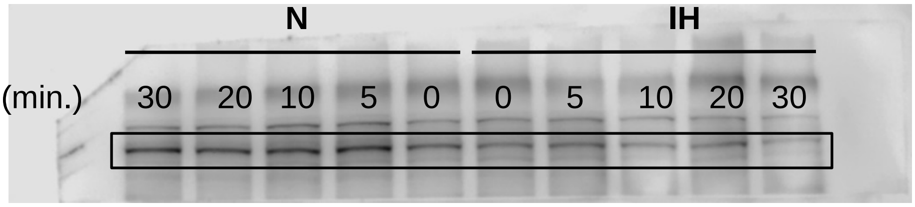

Fig 3a, pAKT

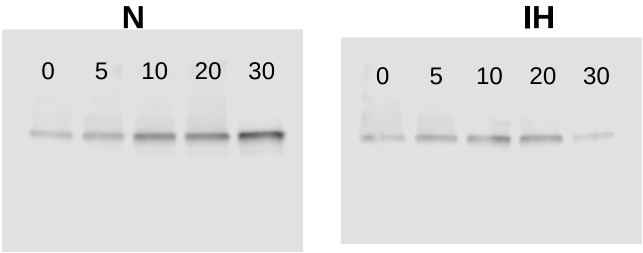

Fig 3a, AKT

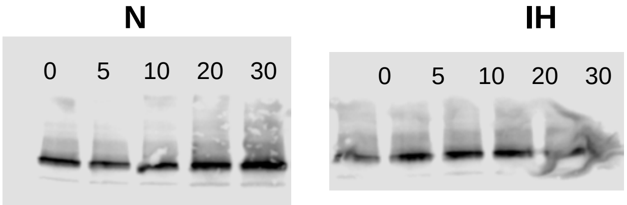

N

(min.) 30 20 10 5 0

Fig 3c: pERK

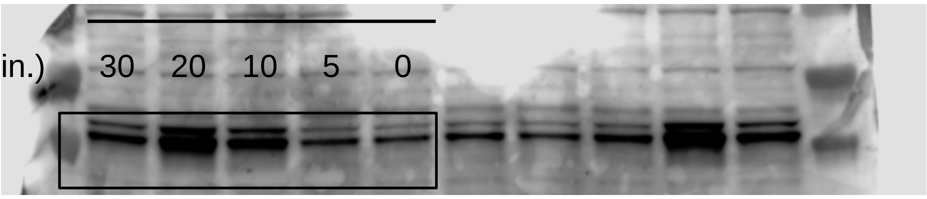

N

(min.) 30 20 10 5 0

Fig 3c, ERK

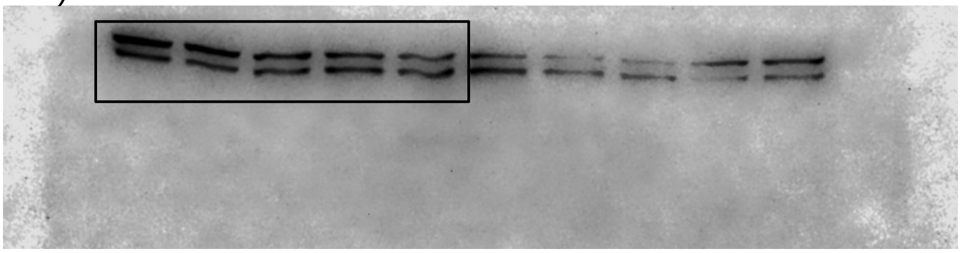

IH

(min.) 0 5 10 20 30

Fig 3c: pERK

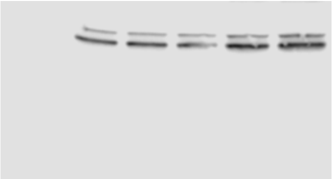

IH

(min.) 0 5 10 20 30

Fig 3c, ERK

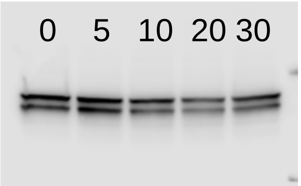

Fig 5

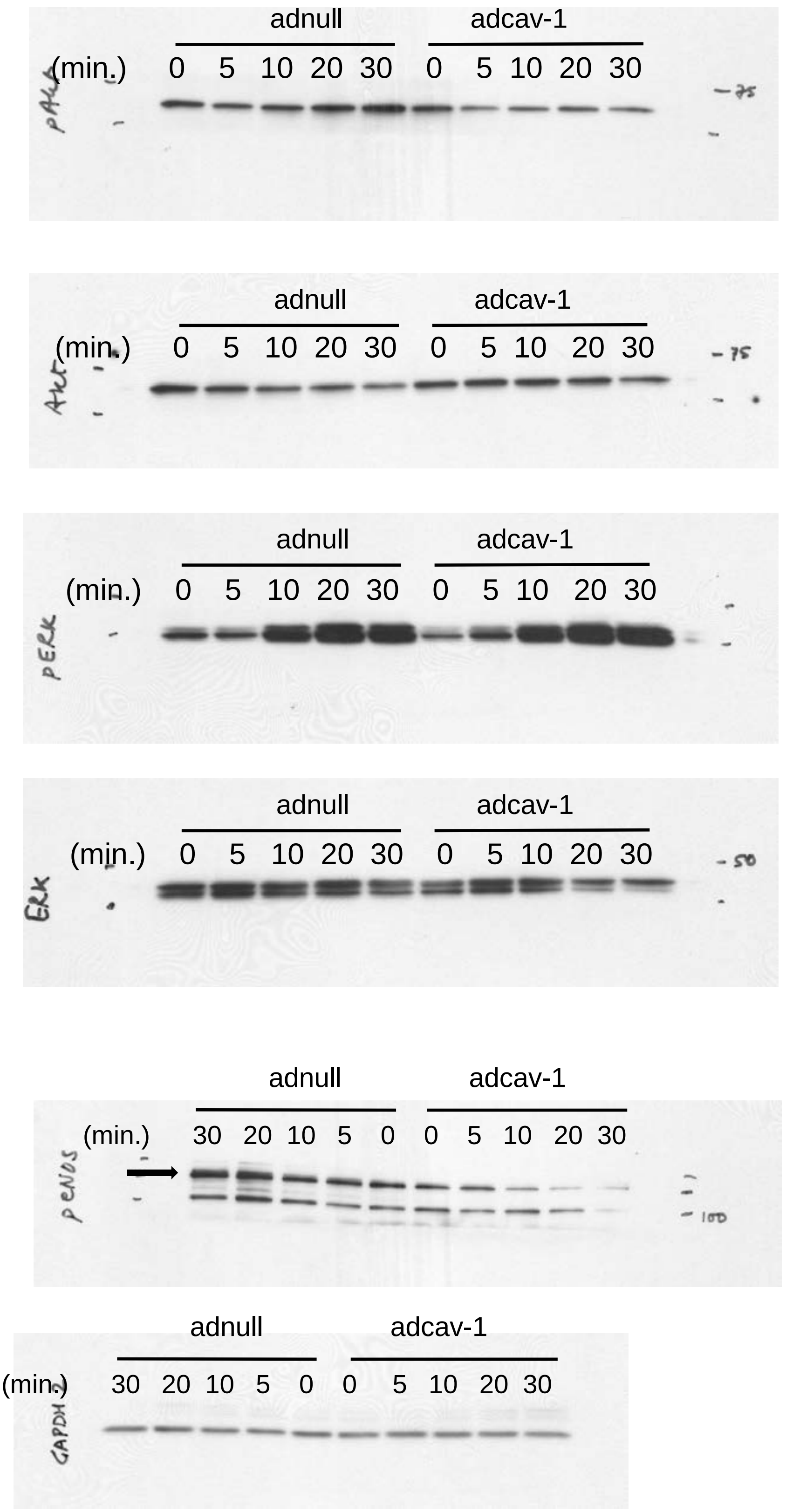
adnull
adcav-1
(min.)
0 5 10 20 30 0 5 10 20 30
adnull
adcav-1
(min.)
0 5 10 20 30 0 5 10 20 30
adnull
adcav-1
(min.)
0 5 10 20 30 0 5 10 20 30
adnull
adcav-1
(min.)
0 5 10 20 30 0 5 10 20 30
ERK
adnull
adcav-1
(min.)
30 20 10 5 0 0 5 10 20 30
adnull
adcav-1
(min.)
30 20 10 5 0 0 5 10 20 30
GAPDH
